# Volume kinetics of lactated Ringer's solution in adult horses

**DOI:** 10.1111/evj.14534

**Published:** 2025-05-13

**Authors:** William W. Muir, Xiu Ting Yiew, Shane W. Bateman, Robert G. Hahn

**Affiliations:** ^1^ College of Veterinary Medicine at the Lincoln Memorial University Harrogate Tennessee USA; ^2^ Ontario Veterinary College at the University of Guelph Guelph Ontario Canada; ^3^ Department of Anesthesiology and Intensive Care Karolinska Institute at Danderyds Hospital Stockholm Sweden

**Keywords:** crystalloids, fluid disposition, fluid therapy, haemodynamics, horse, volume kinetics

## Abstract

**Background:**

Fluid therapy in horses is primarily empirical. Evidence‐based quantification of the disposition of intravenous (IV) crystalloids used in clinical practice could enhance the effectiveness of fluid therapy.

**Objectives:**

To determine the pharmacokinetics (i.e., volume kinetics: VK) and associated haemodynamic effects of IV lactated Ringer's solution (LRS) in adult euvolemic horses.

**Study Design:**

Unmasked multiple subjects, single baseline design.

**Methods:**

Thirteen healthy, euvolemic adult female horses were administered an IV bolus of LRS and monitored over 4 h. Haemoglobin, albumin and haematocrit concentrations were used to generate VK parameter estimates through a non‐linear mixed effects model and stepwise covariate testing. Cardiorespiratory effects, hormonal parameters and urine output were monitored.

**Results:**

Administration of an IV bolus of LRS increased heart rate and systolic arterial pressure. Kinetic analysis was based on plasma albumin, as fluctuations in haemoglobin concentration suggested splenic recruitment of erythrocytes. Fluid disposition was best described by a two‐volume fluid space model. Covariate analysis showed that a high systolic arterial pressure is associated with a decrease in urine output, and that haemoglobin recruitment was associated with the transfer of fluid from the central compartment, which was estimated to be 26.2 L, to a peripheral space. Kinetic constants showed rapid fluid distribution to the peripheral compartment and slow return to the central compartment, impeding fluid elimination from the body. Distribution of LRS from the central compartment was rapid but elimination from the body was slow.

**Main Limitations:**

Limited sample size and sample collection duration may have influenced model selection and covariate identification.

**Conclusions:**

Volume kinetics provides a method for quantitatively describing the volume expanding effects of administered fluids. Fluid infusion is associated with an increase in heart rate and arterial blood pressure. Volume kinetic analysis offers a context‐dependent method for developing and refining more effective fluid infusion protocols.

## Zusammenfassung

**Hintergrund:**

Die Infusionstherapie bei Pferden erfolgt in erster Linie auf empirischer Basis. Eine evidenzbasierte Quantifizierung der Dosierung der in der klinischen Praxis verwendeten intravenösen (IV) kristalloiden Flüssigkeiten könnte die Wirksamkeit der Infusionstherapie verbessern.

**Ziel der Studie:**

Ziel der Studie war es, die Pharmakokinetik (d.h. die Volumenkinetik: VK) und die damit verbundenen hämodynamischen Auswirkungen der intravenösen Verabreichung von laktierter Ringerlösung (LRS) bei adulten euvolemischen Pferden zu bestimmen.

**Studiendesign:**

Unverblendete Studie, mehrere Probanden und einzelner Ausganswert.

**Methodik:**

Dreizehn gesunde, euvolemische, adulte weibliche Pferde wurden nach der Verabreichung von einem IV‐Bolus von LRS für vier Stunden überwacht. Hämoglobin‐, Albumin und Hämatokrit‐Konzentrationen wurden zur Erstellung von VK‐Parameter‐Schätzungen mittels eines Modells mit nichtlinear gemischten Effekten und schrittweiser Kovariatenprüfung verwendet. Kardiorespiratorische Effekte, hormonelle Parameter und die Urinausscheidung wurden überwacht.

**Ergebnisse:**

Die Verabreichung eines IV‐Bolus von LRS erhöhte die Herzfrequenz und den systolischen arteriellen Blutdruck. Die kinetische Analyse basierte auf Plasmaalbumin, da Schwankungen der Hämoglobinkonzentration auf eine Rekrutierung von Erythrozyten aus der Milz hindeuteten. Die Flüssigkeitsdisposition wurde am besten durch ein zweivolumiges Flüssigkeitsraummodell beschrieben. Die Kovarianzanalyse zeigte, dass ein hoher systolischer arterieller Druck mit einer Abnahme der Urinausscheidung verbunden ist und dass die Hämoglobin‐Rekrutierung mit dem Transfer von Flüssigkeit aus dem zentralen Kompartiment, das auf 26,2 l geschätzt wurde, in einem peripherin Raum verbunden war.

Die kinetischen Konstanten zeigten eine schnelle Flüssigkeitsverteilung in das periphere Kompartiment und eine langsame Rückkehr in das zentrale Kompartiment, was die Flüssigkeitsausscheidung aus dem Körper behinderte. Die Verteilung von LRS aus dem zentralen Kompartiment war schnell, aber die Ausscheidung aus dem Körper war langsam.

**Wesentliche Einschränkungen:**

Der begrenzte Umfang an Probanden und die Dauer der Datenerhebung könnten die Modellauswahl und die Identifizierung der Kovarianten beeinflusst haben.

**Schlussfolgerung:**

Die Volumenkinetik bietet eine Methode zur quantitativen Beschreibung der volumenvergröβernden Wirkung verabreichter Flüssigkeiten. Flüssigkeitinfusionen sind mit einem Anstieg der Herzfrequenz und des arteriellen Blutdrucks verbunden. Die volumenkinetische Analyse bietet eine kontextabhängige Methode zur Entwicklung und Verfeinerung effektiver Flüssigkeitinfusionsprotokolle.

## 1 INTRODUCTON

Intravenous (IV) fluid therapy in horses is based upon signalment, history, physical examination, convention, experience and the horizontal application of evidence from other species (e.g., dogs, humans). Whether evidence obtained in one species should be quantitatively extrapolated to the horse is questionable and arguably inappropriate due to known anatomical, physiological, dietary and behavioural differences.^1^, ^2^, ^3^ Intravenous fluids can produce beneficial or harmful effects (e.g., oedema, gut stasis, impaired healing, decreased microvascular blood flow) depending on the dose, rate, frequency of fluid administration, type of fluid administered and the subject's fluid responsiveness.^4^, ^5^ A recent review summarising current concepts for fluid administration in horses highlighted a tendency to overtreat (i.e., excessive rates and volumes) horses, regardless of cost or the lack of evidence for beneficial effects.^6^ Evidence from human medicine suggests that the risks of overzealous fluid resuscitation or maintenance outweigh the benefits.^7^, ^8^, ^9^

Intravenous fluids function like drugs, producing dose‐dependent physiologic effects and side effects.^10^ Pharmacokinetic (PK) compartmental modelling, and specifically volume kinetics (VK) describes the disposition of administered fluids in the body.^11^, ^12^ Volume kinetic models are determined by monitoring serial concentration‐time changes in an appropriate plasma biomarker. Haemoglobin and albumin, with their relatively slow turnover rates and acute changes following IV fluid administration, are suitable for analysing body fluid compartment changes.^11^, ^12^ Intravenous fluids are initially distributed throughout a central compartment (*V*_*c*_), which often corresponds to the plasma volume (PV), before moving into one or more peripheral compartments (*V*_*p*_ or *V*_1_, *V*_2_).^11^ Fluid exchange between compartments is governed by hydrostatic and osmotic forces and membrane permeability.^13^, ^14^ Fluid VK parameters (e.g., half‐life, volume of distribution, elimination) describe the disposition of infused fluids and can be used to develop safer and more effective dosage regimens.

Despite the routine administration of IV fluids in clinical practice, no studies describe their disposition in healthy adult horses. We hypothesised that VK modelling could effectively describe the IV disposition of lactated Ringer solution (LRS) in healthy adult horses. The primary objectives of our study were to determine the volume kinetic, cardiorespiratory and blood chemical effects (i.e., atrial natriuretic peptide: ANP; aldosterone, copeptin) produced by an IV LRS bolus in healthy adult horses.

## 2 MATERIALS AND METHODS

### 2.1 Horses and examination

Thirteen female horses, aged 4–16 years and weighing 400–560 kg (463 ± 56 kg), were enrolled. Each horse was deemed healthy based on physical examination, complete blood count, serum biochemistry and urinalysis. The horses were housed in individual stalls and acclimated to the facility and personnel for up to 10 days before the experiment. They were fed an 11% protein commercial horse concentrate (Co‐Op: LaVergne) in quantities totalling approximately 0.5% of bodyweight twice daily. Grass or alfalfa hay was provided daily, totalling approximately 1.5% to 2% of bodyweight. All horses had free access to fresh water and were exercised daily throughout the acclimation period. The horses for this study were selected from a larger pool of horses, had not been used in a previous study, were familiar with personnel, and were conditioned to their environment and data collection procedures prior to the experiment. Vital sign baseline values were like those obtained during daily unstressed health checks.

### 2.2 Study design

An unmasked, single‐baseline, multiple‐subject before and after exploratory study was designed. A low‐stress environment was maintained throughout the experiment by minimising the number of personnel, environmental activity and unnecessary or disruptive (e.g., loud) sounds. Previous studies in humans suggested that a minimum of 10 subjects was necessary to characterise the VK of an LRS fluid infusion.^15^ A power calculation based upon mean and standard deviation of arterial blood pressure suggested that a minimum of 12 horses was needed to detect a change with 95% confidence.

### 2.3 Experimental procedures and instrumentation

Each mare served as its own control and all data and sample collection time points were made in relation to baseline (time 0, beginning of LRS infusion). Each mare was administered butorphanol 0.01 mg/kg IV and midazolam 0.01 mg/kg IV approximately 45 min before IV LRS administration. Local anaesthetic (2% lidocaine) infiltration and standard aseptic skin preparation preceded IV catheter placement. A 16 G × 21 cm central venous catheter with a catheter lumen volume of 0.42 mL (Mila International) was advanced into the left jugular vein to the level of the right atrium. An extended‐use 12 G × 13 cm IV catheter (Mila International) was positioned in the right jugular vein. A urinary Foley catheter (Jorvet) was advanced into the bladder.

Three baseline (time 0) blood samples were collected 1–3 min before IV LRS infusion for determination of haematocrit (Hct), blood haemoglobin (B‐Hb), plasma albumin (Alb) and plasma creatinine (Cr). Each mare was administered 20 mL/kg LRS IV (Dechra Veterinary Services) over 30 min via the right jugular vein using a large animal solution administration set (STAT Large Animal IV System, International WIN, Ltd.) and fluid infusion system (NE‐9000Bc: New Era Pump Systems Inc.). Blood samples were collected from the central venous catheter using a modified three‐syringe technique^16^: instillation of 5 mL of saline flush followed by several aspirations of a minimum of 5 mL of a diluted blood sample (syringe 1), collection of a 5 mL blood sample (syringe 2), then return of initial diluted blood sample (syringe 1) followed by an additional 5 mL of saline flush (syringe 3). The five (5.0) mL of whole blood sample was collected from the left central venous catheter into ethylenediaminetetraacetic acid (EDTA) tubes, with a total of 5.0 mL of LRS flush returned to the horse.

Blood samples for determination of B‐Hb (HemoCue)^17^ and Alb (Alb; Cobas 6000 analyser series with the Cobas c 501 module) concentrations were measured at baseline (time 0), every 5 min during infusion for 60 min (12 samples), and at 70, 90, 110, 130, 150, and 180 min post‐infusion (18 total blood samples). Plasma creatinine concentration was determined (Cobas 6000 analyser series with the Cobas c 501 module) at baseline (time 0) and between 6 and 8 h after beginning LRS infusion. The bladder was emptied 5 min before IV fluid administration. An empty bladder was confirmed before LRS infusion by applying negative pressure to a 60 cc syringe and repositioning the urinary catheter until no urine could be withdrawn. Urine volume was recorded at 30‐min intervals (30, 60, 90, 120, 150, 180, 210 and 240 min) for 4 h after initiating IV LRS administration. Urine samples were pooled to determine the total 4‐h urine volume. Urine osmolality, creatinine and albumin concentrations were determined at baseline (time 0) and 4 h after beginning (time 0) IV LRS administration.

Plasma copeptin (i.e., a stable surrogate marker for vasopressin), atrial natriuretic peptide and aldosterone (Equine specific ELISA kits: Biovenic Inc.; Figure S1) concentrations were determined from 10 of the 13 horses at baseline (time 0) and at 0.5, 1 and 8 h after beginning IV LRS administration. Blood samples were stored at −80°C for post‐study analysis. Respiratory rate (RR: breaths/min) was determined by auscultation. Heart rate (HR: beats per min), systolic (SAP), diastolic (DAP) and mean (MAP) indirect arterial blood pressure (mm Hg), and body temperature (°F) were monitored (Vet 30E, SunTech Medical, Inc.) and recorded coincident with blood sample (i.e., B‐Hb and Alb) collections. Intravenous and urinary catheters were removed after the 4‐h data collection period, and the horses were returned to their stalls.

### 2.4 Volume kinetic (VK) analysis

The mean of triplicate baseline B‐Hb, Alb and Hct measurements was averaged and used as initial baseline values for each individual horse. Plasma concentrations of B‐Hb, Alb and Hct at baseline (time 0) were set to zero. Plasma dilution at each time point was calculated from serial Alb concentration determinations using computer software (Microsoft Excel, Microsoft Canada). The formula used to calculate serial plasma dilution from uncorrected albumin concentration was ((Alb_0_‐Alb_*t*_)/Alb_*t*_) where Alb_0_ and Alb_t_ are the albumin concentrations at time *t* = 0 and *t* = elapsed time since *t* = 0, respectively.

Population maximum likelihood modelling with rate constant parameterization was performed using the Naïve Pooled algorithm and optimised with the First‐Order Conditional Estimation with Extended Least Squares (FOCE‐ELS) algorithm, using a commercial pharmacokinetic analysis software package (Phoenix 8.4, Certara) (Code S1). Separate fittings were conducted for one‐volume fluid space (1‐VOFS), two‐volume fluid space (2‐VOFS) and three‐volume fluid space (3‐VOFS) kinetic models. Input variables included mean plasma dilution, LRS infusion rate, time, subject identification and measured cumulative urine output. Best parameter estimates ($V_{c}$, $k_{10}$, $k_{12}$, $k_{21}$, $\;k_{23}$, $k_{32}$) were generated from these inputs. The statistical fit of the 1‐VOFS, 2‐VOFS and 3‐VOFS kinetic models was compared. The base model for subsequent covariate analysis was selected based on the lowest Akaike Information Criterion (AIC) value, if parameter estimates were realistic and had coefficients of variation (CV%) <50%.

Fluid is infused at the rate *R*_*o*_ into a central volume (*V*_*c*_, the plasma) in the 2‐VOFS kinetic model, from where distribution occurs at a rate (*k*_12_) to the extravascular space (*V*_*p*_). Redistribution of fluid from *V*_*p*_ to *V*_*c*_ occurs at a rate governed by a second rate constant (*k*_21_, lymphatic flow). The measured urine output divided by the modelled volume expansion of *V*_*c*_ yielded the elimination rate constant (*k*_10_).

The rate of flow between the compartments at any time (mL/min) is given by the product of the rate constant and the volume expansion of the fluid space from which the flow started. The differential equations describing the model are:

$$dv_{c}/dt=R_{o}–k_{12}\;\left(v_{c}–V_{c}\right)+k_{21}\;\left(v_{p}–V_{p}\right)–k_{10}\;\left(v_{c}–V_{c}\right),$$

$$dv_{p}/dt=k_{12}\;\left(v_{c}–V_{c}\right)–k_{21}\;\left(v_{p}–V_{p}\right),$$

$$dU/dt=k_{10}\;\left(v_{c}–V_{c}\right),$$

where the capital letters denote the baseline volumes of each fluid compartment, and the lower‐case letters represent the expanded volumes. *U* is the urine flow rate.

Haemoglobin recruitment was obtained by taking the difference between the Hb‐derived and albumin‐derived haemodilution. Derived blood Hb is calculated as [BV_o_ × Hb_o_/Hb_t_] where BV_o_ is the baseline blood volume and equals 8% of the bodyweight. The blood haemoglobin concentration (Hb) at baseline has the subscript o and Hb measured a later time the subscript t. The corresponding equation for the haemodilution using plasma albumin is written [BV_o_ × (1‐Hct_o_) × Alb_o_/Alb_1_/(1‐Hct_o_)]. These equations should show the same result if no Hb is recruited. First, the (1‐Hct_o_) expression transforms BV to PV and, after multiplication with the plasma dilution, converts the entire expression back to BV. A difference between them (as the weight of Hb, g) was obtained as Hb_t_ × (albumin dilution – Hb dilution). Covariates that could refine the VK base model were identified by reviewing the effects plots graphed by the Phoenix program. Potential covariates were SAP, DAP, HR, RR, temperature, bodyweight and haemoglobin recruitment. Covariate analysis was performed using the stepwise covariate search function (forward addition and backward elimination) in the software, with thresholds set at *p* = 0.05 for adding and *p* = 0.01 for removing a covariate effect. A covariate effect was added to the model if it reduced the negative 2 Log Likelihood (−2LL) by more than the critical value of 3.84 (*p* < 0.05) and was removed from the model if it increased the −2LL by less than the critical value of 6.64 (*p* < 0.01). A covariate effect was considered statistically significant if the confidence interval for the parameter estimate did not include 0.

The distribution half‐life was obtained by simulating the central volume–time curve over time and taking the time required for half of the volume decrease between the end of the infusion and the terminal expansion. Likewise, the half‐life of the fluid in the body was obtained by simulating the volume expansion of both the central and the peripheral compartments and observing the time required for their sum to decrease by 50%. This calculation was started after distribution was complete (i.e.,100 min). The mean residence time (MRT) for LRS was MRT = 1.44 × half‐life.

The pharmacokinetic software's (Phoenix 8.4, Certara) simulation function was used to perform fluid simulations. These simulations aimed to demonstrate the effects of covariates and suggest fluid administration strategies based on the parameter estimates identified in the final VK model.

### 2.5 Data analysis

A one‐way ANOVA followed by Dunnett's comparison test was used to determine differences in temperature, RR, HR, blood pressure and Cr levels (GraphPad Prism 5). B‐Hb, Hct, plasma albumin, heart rate, RR, temperature, arterial blood pressure, measured urine output and hormone (i.e., copeptin, atrial natriuretic peptide, aldosterone) concentrations are reported as mean ± standard deviation and 95% confidence interval (CI). A *p*‐value of <0.05 was considered statistically significant. Volume kinetic parameters are reported as the optimal estimate, standard error, 95% CI and the coefficient of the between‐subject variation (CV%).

## 3 RESULTS

Thirteen horses completed the experiment. No abnormal physical signs, respiratory patterns or abnormal behavioural changes were recorded during the experiments. One horse objected to being positioned in the stockade and was rescheduled for restudy. Data from all 13 horses were used to derive VK parameters and create simulations. Plasma copeptin, atrial natriuretic peptide and aldosterone concentrations were determined from 10 of the 13 horses. The plasma samples were collected and stored at −80°C for approximately 5 months before assay completion. Approximately 130 mL of blood was collected during the experiment for haematologic (PCV, B‐Hb, Alb,) and blood analyte analysis.

**FIGURE 1:**
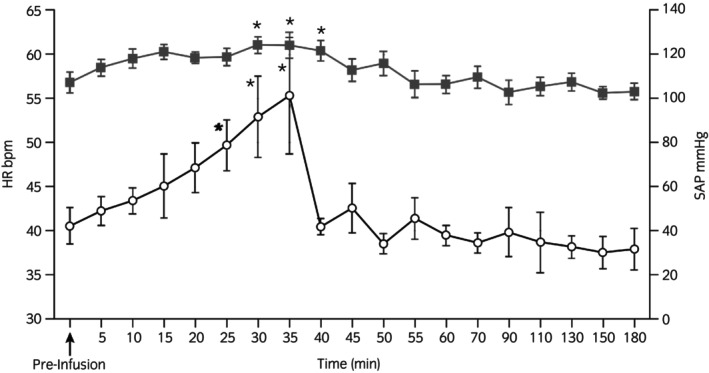
Mean heart rate (HR) and systolic blood pressure (SBP) changes produced by 20 mL/kg IV LRS over 30 min to euvolemic horses. Pre‐infusion (Time 0 min), heart rate (open circles); systolic arterial blood pressure (black squares). *p < 0.05.

The horses received 9.48 ± 1.59 L of LRS over 30 min. The infusion did not change body temperature (37.2°C; CI 37.2–37.5) or respiratory rate (23 ± 4 breaths/min; CI 19–27) from pre‐infusion values. Heart rate increased from pre‐infusion values during the 30‐min LRS infusion from 42 ± 7 bpm (CI 37–35) to a peak value of 53 ± 16 bpm (CI 37–69). Systolic, diastolic and mean arterial blood pressure increased from 107 ± 16 (CI 98–126) to 124 ± 14 (CI 117–131) mm Hg, 66 ± 17 (CI 57–75) to 76 ± 9 (CI 71–81) mm Hg and 79 ± 16 (CI 70–88) to 92 ± 10 (87–97) mm Hg, respectively, during the LRS bolus infusion (Figure 1).

The LRS infusion initially decreased Hct from 36 ± 3 (CI 34–38) to 31 ± 3 (29–33) %, B‐Hb from 12.3 ± 1.4 (CI 11.5–13.1) to 11.2 ± 1.0 (CI 10.6–11.8) g/dL and Alb from 3.2 ± 0.3 (CI 3.0–3.4) to 2.7 ± 0.2 (CI 2.6–2.8) g/dL. Blood haemoglobin concentrations increased above baseline values at 35 to 50 min (12.9 ± 0.9 g/dL) after starting the LRS infusion in six of 13 horses, making it an unreliable index of plasma dilution. Plasma Cr (88.42 ± 8.84 μmol/L), urine osmolality (1012 ± 318 mOsm/kg; CI 835–1189), urine albumin (<0.7 g/L) and urine creatinine (10,787 ± 4067 μmol/L; CI 8488‐13,086) did not change from baseline values. Urine production (0.62 ± 0.3 mL/kg/day; CI 0.61–0.63) was uniformly low compared with normal values reported for adult healthy mares (0.61 to 1.1 mL/kg/h).^18^ Plasma copeptin (4.8 ± 1.4 pg/mL; 95% CI 4.0–5.7), atrial natriuretic peptide (136.2 ± 18.2 pg/mL; 95% CI 124.9–147.5) and aldosterone (159.3 ± 12.0 pg/mL; 95% CI 151.9–166.7) did not change from baseline values.

### 3.1 Volume kinetics

**FIGURE 2:**
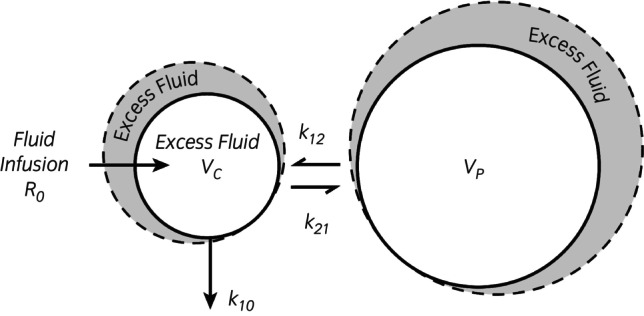
Two‐volume fluid space kinetic model. Central compartment (*V*_*c*_); peripheral compartment (*V*_*p*_); infusion rate (*R*_*o*_); central to peripheral distribution rate constant (*k*_12_); peripheral to central redistribution rate constant (*k*_21_); elimination rate constant (*k*_10_). *R*_*o*_ = 20 mL/kg LRS IV over 30 min for these experiments. The size of the peripheral space (*V*_*p*_), obtained as *V*_*c*_ × *k*_12_/*k*_21_, is supraphysiological, which indicates the absence of free flow.

The VK analysis was based on 252 measurements of the albumin‐based plasma dilution and 92 measurements of the urine output. Uncorrected albumin‐based plasma dilution data from all 13 horses were fitted to 1‐VOFS, 2‐VOFS and 3‐VOFS kinetic models. The 2‐VOFS kinetic model produced the lowest Akaike Information Criterion (AIC) score (519.8) indicating the lowest uncertainty and best statistical fit (Figure 2; Table S1). Covariate analysis identified a negative influence of SAP on *k*_10_ and a positive influence of haemoglobin recruitment on *k*_12_. The final model parameter estimates, including significant covariate effects, are shown in Table 1. Deviations of data from predicted values was 0.094 for the base model and 0.096 for the final model, refuting over‐parametrisation. The distribution half‐life from *V*_*c*_ was 15.0 min and the half‐life for the infused volume in the body was 75.8 h, corresponding to a mean residence time of 109 h.

**TABLE 1:** Volume kinetic (VK) parameter estimates for the statistically justified VK model of lactated Ringer's solution analysis in conscious, euvolemic healthy adult horses.

Parameter	Estimate	Standard error	CV%	2.5% CI	97.5% CI
*V*_*c*_, volume of central fluid space (mL)	26,202	2411	9.20	21,458	30,946
*k*_10_ (× 10^−3^ min^−1^): Elimination rate constant (/min)	2.11	0.55	26.2	1.02	3.21
*k*_12_ (× 10^−3^ min^−1^): Central to peripheral distribution rate constant (/min)	51.4	5.4	10.4	40.8	61.9
*k*_21_ (× 10^−3^ min^−1^): Peripheral to central redistribution rate constant (/min)	4.15	1.47	35.4	1.25	7.05
Effect of SAP (mm Hg) on *k*_10_, covariance	−4.58	1.2	−26.1	−6.93	−2.23
Effect of Hb (g/dL) recruitment (splenic contraction) on *k*_12_, covariance	0. 71	0.2	21.0	0.41	0.99

*Note*: Equations: *k*_10_ = 2.11 × [(SAP/112.5)^−4.58^] × 10^−3^; *k*_12_ = 51.4 × [(recruited Hb/152)^0.71^] × 10^−3^.

**FIGURE 3:**
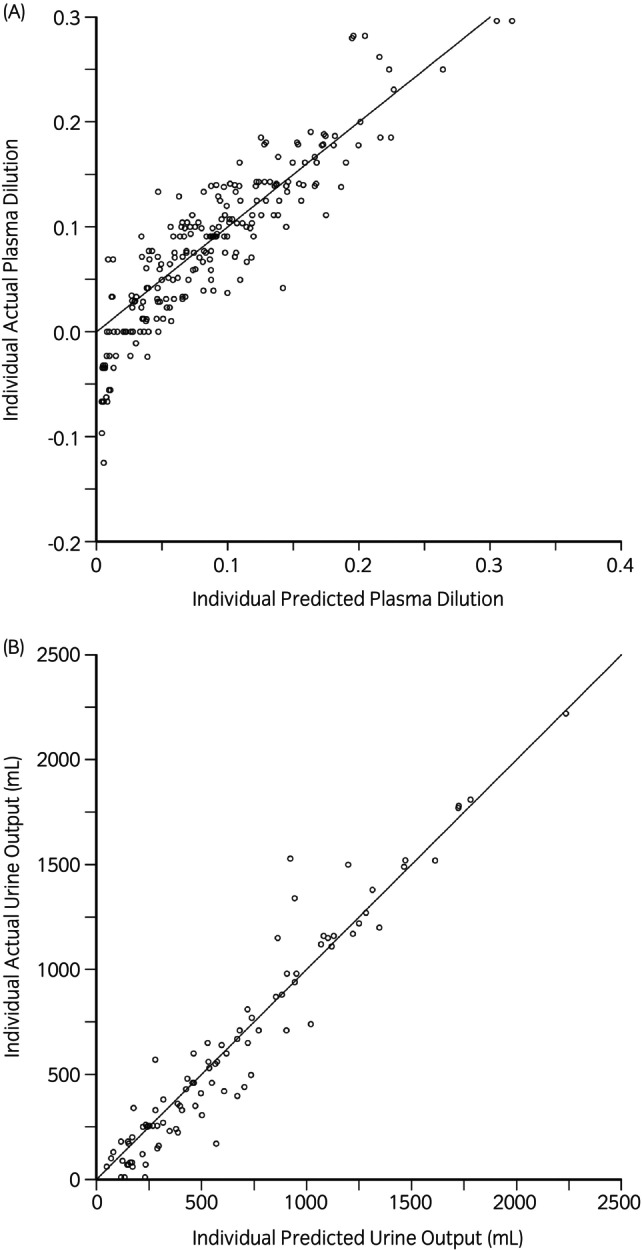
(A) Agreement between the model‐predicted plasma dilution and observed values, with significant covariates included. (B) Agreement between the model‐predicted urinary excretion and observed values, with significant covariates included. The solid black line in A and B indicates perfect agreement.

Model‐predicted values for plasma volume and urine output, as depicted in the actual versus predicted plots, demonstrated improved fit with observed values after accounting for the two significant covariates (i.e. SAP and haemoglobin recruitment) (Figure 3). Plots of weighted residuals also showed improved model fit with the inclusion of these covariates (Figure S2). Visual Predictive Checks of observed versus model‐predicted plasma dilution and urine output further supported an acceptable model fit (A).

**FIGURE 4:**
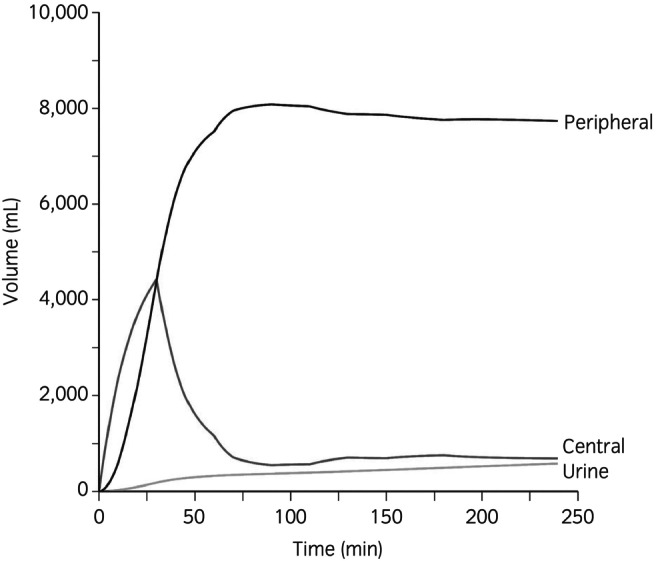
Distribution of 20 mL/kg of infused lactated Ringer's solution administered over 30 min in conscious, euvolemic adult horses.

**FIGURE 5:**
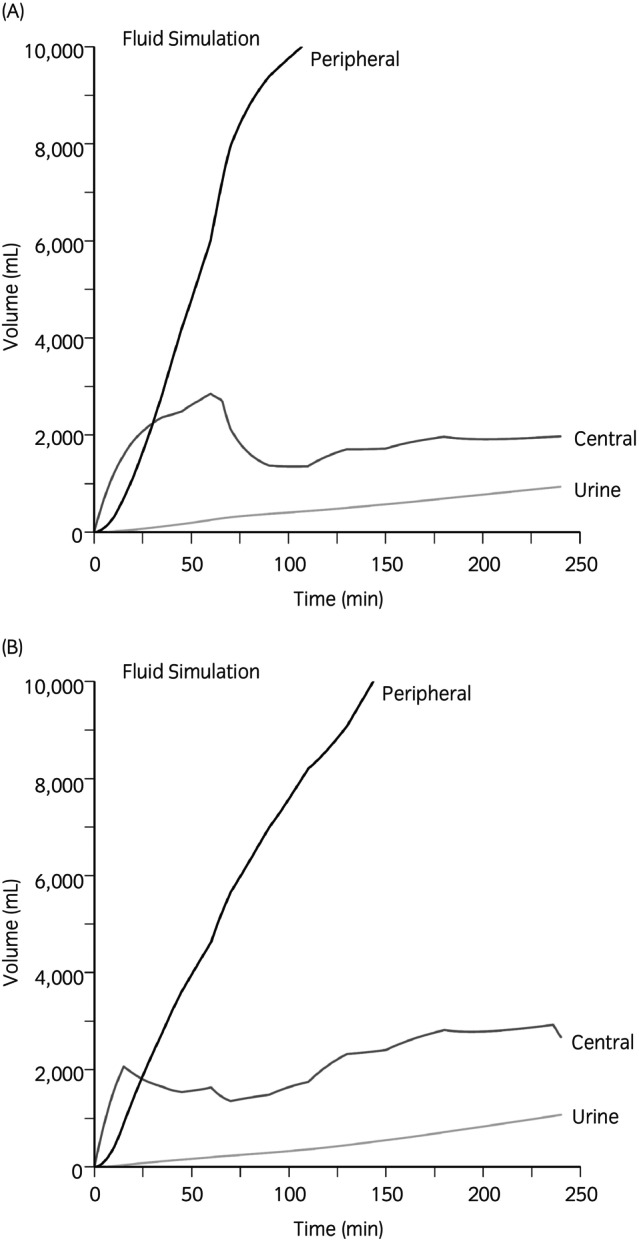
Simulation in a 450 kg horse. (A) Simulation of a 10 L bolus over 1 h (22 mL/kg) followed by a 5 mL/kg/h infusion of lactated Ringer's solution (LRS) with systolic blood pressure (SAP) arbitrarily set at 111 mm Hg. (B) Simulation of a 3 L bolus over 15 min (6.6 mL/kg) followed by a 9 mL/kg/h infusion of LRS with systolic blood pressure arbitrarily set at 111 mm Hg. Note the rapid accumulation of fluid in the peripheral compartment in A.

Simulations were performed to illustrate the distribution of 20 mL/kg of IV LRS administered over 30 min (Figure 4). Unexpectedly, increased SAP reduced fluid elimination, promoting fluid accumulation in the peripheral space (Figure 5). The impact of haemoglobin recruitment on the *k*_12_ rate constant was less pronounced. Derived parameter estimates from the VK analysis (Table 1) were inserted into the simulation module of the Phoenix software and the fluid distribution following a single 60‐min and a two‐stage 15‐min infusion were plotted (Table 2).

**TABLE 2:** Fluid rates required to produce (0–30 min) and maintain (30–60 and 60–180 min) an expected plasma volume expansion of 5%, 10% or 15% over 3 h in a 450 kg healthy horse.

	Fluid infusion (mL/min)			Volume increase (L)	Volume infused (L)	V_*p*_, expansion (L) at 80 min	Urine (L) at 180 min
*V*_*c*_ (%) expansion	0–30 min	30–60 min	60–180 min				
5%	50	35	25	0.75	5.5	4.5	0.26
10%	100	75	50	1.5	11.3	9	0.52
15%	150	100	75	2.25	16.5	13.4	0.76

*Note*: The initial infusion rate needs to be adjusted twice to create and maintain a steady state. The fluid infusion simulations are based on data from Table 1. Times represent duration of infusion.

## 4 DISCUSSION

The results demonstrate that infused LRS is slowly eliminated in horses. The rate of distribution is the same, but the return flow of distributed fluid (*k*_21_) and diuretic response to fluid loading (*k*_10_) are only 15%–20% of what is reported in awake adult humans.^10^, ^11^ The slow turnover of fluid is congruent with the ascribed tolerance to water deprivation and sweating in horses. Interestingly, the fluid retention became more intense when blood pressure was high, which is opposite to what is found in humans.^11^ Our study also, for the first time, identified and clarified the fluid kinetic consequences of Hb recruitment from the spleen, which increases the oxygen carrying capacity of the blood and is important during strenuous physical exercise.

### 4.1 Volume kinetics in horses

Our study is the first to report the VK of IV LRS in conscious horses. Traditional PK methods use repeated measurements of a drug's plasma concentration‐time course to establish its disposition (i.e., distribution, elimination) in the body, generating PK parameters (i.e., half‐life, volume of distribution, clearance) that inform therapeutic protocols. Volume kinetics uses changes in plasma concentration‐time data (i.e., dilution) of a naturally occurring plasma constituent to determine the disposition of an administered fluid and generate VK parameters that guide fluid administration.^11^, ^12^ The monitored plasma constituent must be stable, easily detectable and acceptably impervious to capillary leakage.^11^ Both B‐Hb and plasma Alb fulfil these criteria in humans, sheep, pigs, cats and horses and are used to determine blood and plasma volumes, deriving VK parameters for both crystalloid and colloid solutions in conscious and anaesthetised mammals.^11^, ^12^, ^19^, ^20^, ^21^ Haematological and performance studies in resting and exercised horses have verified that the equine spleen is a dynamic red blood cell reservoir that is sensitive to rapid changes in plasma red blood cell concentration.^22^, ^23^, ^24^, ^25^, ^26^, ^27^ Splenic contraction, which is sympathetically mediated, can increase the horse's total circulating red blood cells (i.e., Hct) by more than 30%.^23^, ^25^, ^26^, ^27^ The rapid infusion of LRS in the current study triggered splenic contraction in some horses, rendering Hb determinations unsuitable for VK analysis. This finding was unexpected and requires further investigation since the effect of IV fluid administration (e.g., rate, volume) on splenic contraction in horses is unknown. Previous studies in horses have shown that splenic contraction does not affect plasma Alb concentration, supporting its use as a suitable marker for VK analysis in healthy euvolemic horses.^11^, ^25^, ^26^

Volume kinetic analysis of LRS infusion using albumin‐derived plasma dilution data generated a *V*_*c*_ of 26.2 ± 2.4 L, corresponding to 5.6% of bodyweight. This volume is consistent with values reported in studies using alternative techniques (e.g., Evans blue dye, radioactive iodine) to determine PV in horses.^24^, ^28^, ^29^, ^30^, ^31^ A 2‐VOFS kinetic model best represented LRS disposition in horses. Although a 3‐VOFS kinetic model was also considered, it did not produce stronger evidence (i.e., lower AIC) for acceptance. Goodness‐of‐fit plots from group analysis identified good agreement between the observed experimental data and model predictions, further supporting the selection of the 2‐VOFS model (Table S1). However, the parameter estimate results in this study suggest that marked differences in fluid disposition exist among species, confirming that species‐specific fluid administration guidelines should be developed.^11^, ^12^

The present study identified arterial blood pressure and splenic contraction as significant covariates to be included in the final model. Notably, increases in arterial blood pressure were associated with a decrease in fluid elimination from the body (*k*_10_). This negative relationship was unexpected, as previous studies in humans and animals have shown a positive correlation (i.e., pressure‐induced diuresis).^28^, ^32^, ^33^ In addition to fluid infusion, splenic contraction may have facilitated the rate of fluid movement from the central to peripheral compartment (i.e., *k*_12_) in our horses, presumably due to the additional volume of blood entering the circulation from the contracting spleen, coupled with the increase in systemic arterial blood pressure. Further study is required to determine the effects of infusion rate, volume, and the effects of splenic contraction on these findings.

### 4.2 Distribution of infused fluid

Contemporary views regarding the disposition of crystalloid solutions (e.g., LRS) propose that more than 75% of an infused crystalloid is rapidly distributed into a peripheral compartment, with volume‐expanding effects reduced to less than 20% within 30 min post‐infusion.^34^ In our study, the infusion of 20 mL/kg LRS over 30 min in horses increased *V*_*c*_ by approximately 4.4 L, indicating that almost 50% of the infused fluid (20 mL/kg × 450 kg = 9000 mL; 4.4 L ÷ 9 L = 0.49) remained in the vascular compartment at the end of the infusion. This volume decreased to less than 10% (0.7 L ÷ 9 L = 0.08) 30 min after the end of the LRS infusion, indicating a rapid transfer of fluid (i.e., large *k*_12_) out of *V*_*c*_.^11^ However, the rate constant for fluid return to *V*_*c*_ (*k*_21_) in our horses was 15%–20% of the rate found in conscious humans and only 2% of the value reported for conscious cats.^11^, ^35^, ^36^ Additionally, fluid elimination (*k*_10_) in horses was only 15%–20% of what has been measured in humans but similar to the cat.^35^, ^36^ This difference might be attributed to species differences or an inadequate sample size which may have been insufficient to reveal the existence of a “third fluid space”.^37^ This third fluid compartment becomes apparent in humans with larger fluid infusions (>20 mL/kg) and is characterised by a very low turnover rate.^35^ The large *k*_12_ value, combined with comparatively low *k*_21_ and *k*_10_ values in our healthy euvolemic horses, suggests that a large proportion of rapidly administered IV fluid is retained in a peripheral compartment. This fluid retention is likely to produce tissue oedema when large fluid volumes are administered or if the horse is nonresponsive (i.e., does not show improvement) to IV fluid therapy.^38^, ^39^ As previously noted, fluid elimination (*k*_10_) did not increase with the arterial blood pressure, unlike in other species evaluated to date.^28^ Horses have adapted to periods of water restriction by accumulating water in their hindgut, similar to camels, goats and desert sheep.^40^ This hindgut water accumulation may be teleologically advantageous, helping to preserve plasma volume (e.g., entero‐systemic cycle) during periods of water deprivation.^2^, ^41^, ^42^ The long redistribution time from the peripheral to the central compartment (i.e., comparatively low *k*_21_) in our horses corroborates previous concerns and current speculations that, unlike humans, horses can accumulate IV administered fluids in a peripheral compartment or reservoir.^2^, ^6^, ^42^

### 4.3 Simulations

Volume kinetic data were used to simulate fluid infusion‐induced volume expansion. These simulations offer the opportunity to develop ideal dosing regimens that optimise plasma volume expansion while minimising side effects (i.e., fluid accumulation). Overall, these simulations suggested that larger and faster bolus infusions result in greater fluid accumulation in the peripheral (i.e., interstitial) compartment. Conversely, strategies involving smaller fluid boluses followed by bracketed maintenance infusion rates may be more effective in maintaining central compartment volume and achieving the intended clinical goals. Simulations were employed to achieve and maintain predetermined plasma volume expansions (5%, 20%, 15%) in a 450 kg horse by two adjustments of the initial infusion rate (Table 2).

Simulations used power models for the covariances, and the changes are calculated based on the mean values and the exponents. Hence, the value of *k*_10_ refers to the mean value for all measurements in all horses of 112.5 mm Hg. A rise of SAP amounting to 5 mm Hg above the mean value gives the equation 2.11 × ((117.5/112.5)^−4.58^) × 10^−3^, which is 1.73 × 10^−3^, and thus a reduction by 18%.

### 4.4 Physiological responses

Intravenous fluids are administered to increase blood volume, which in turn elevates arterial blood pressure. This rise in pressure is expected to decrease heart rate (i.e., baroreceptor reflex; Mareys Law) by inhibiting sympathetic activity and promoting parasympathetic tone.^43^, ^44^ The magnitude of this response is dependent upon the subject's volume status, level of consciousness, pre‐existing arterial blood pressure, heart rate and neuroendocrine activity.^45^ The infusion of 20 mL/kg IV LRS to conscious horses in the current study paradoxically increased both arterial blood pressure and heart rate, with these changes being most prominent towards the end of the LRS infusion. Intravenous fluid administration has been previously shown to increase venous filling pressure and heart rate in chloroform‐anaesthetised dogs (i.e., atrial stretch: Bainbridge reflex).^46^ To our knowledge, this is the first report of the atrial stretch (i.e., Bainbridge) reflex in response to fluid infusion in healthy, conscious, euvolemic horses. A similar response has been observed in conscious humans, dogs and baboons, regardless of increases in systolic blood pressure.^47^ The atrial stretch reflex is mediated by low‐pressure (Type B) stretch receptors located at the junction of the vena cava and pulmonary veins with their respective (i.e., right and left) atria. Increases in venous return and central volume inhibit vagal outflow and enhance sympathetic tone to the sinoatrial node, resulting in an increase in heart rate.^48^ In addition, acute increases in ventricular volume can also trigger a stretch‐induced inhibitory reflex (i.e., Bezold–Jarich reflex) that increases parasympathetic activity and inhibits sympathetic activity.^49^, ^50^ Limited research suggests that the baroreceptor reflex predominates during IV fluid administration in hypovolemic subjects, while the atrial stretch reflex is more prominent in euvolemic subjects and when central volume is increased.^47^, ^48^ The rapid decrease in heart rate after the LRS infusion in the current study strongly suggests baroreceptor reflex activity.

### 4.5 Limitations

This study was conducted in healthy euvolemic horses. This was an exploratory study of limited sample size and model parameters may change with the generation of additional data and a longer sampling period. Pharmacokinetics, VK and compartmental analysis are mathematical methods used to quantitatively estimate, describe and predict the disposition of drugs and fluids within the body. Volumes of distributions are defined by the model and do not necessarily correspond to physiological volumes. These methods and the data they generate are dependent upon physiologic conditions (e.g., cardiac output, organ blood flow, blood pressure, metabolism, renal tubular function, urine production). The main difference between PK and VK is that *V*_*c*_ and *V*_*p*_ can expand or contract and that *V*_*c*_ is very close to the plasma volume, thereby improving its physiologic relevance.

Dilution of plasma albumin concentration has been validated as a dependable determinant of PV in healthy horses, despite minor time‐dependent losses from the circulation. Changes in derived rate constants (*k*_12_, *k*_21_) are likely to represent reduction or expansion in PV in healthy animals, since albumin's reflection coefficient is close to one in most healthy tissues.^51^, ^52^ The potential loss of albumin from the circulation following IV LRS administration could contribute to an increase in the calculated *V*_*c*_, and inflammatory diseases that increase albumin loss from *V*_*c*_ could complicate its use as a relevant biomarker of fluid flux. A longer sampling period would produce a more precise value of the elimination half‐life, but we did not anticipate that the elimination of the LRS would be so slow when the study was planned. A 3‐h collection period is considered adequate in other species.^11^ Finally, studies in humans suggest that *k*_21_ (i.e., re‐distribution) likely represents fluid return to the circulation via the lymphatic system.^53^ Whether *k*_21_ is representative of lymphatic flow in adult horses requires further study. Future studies are required to determine the effects of different fluids (crystalloids, colloids), dosage protocols (rates, volumes) and disease states on derived VK rate constants.

### 4.6 Conclusions and clinical implications

Volume kinetics provides a method for quantitatively describing the volume expanding effects of administered fluids, thereby providing meaningful inferences that can be used to guide fluid therapy. The quantitative parameters and models derived from these methods can be used to develop effective and safer fluid therapy protocols. The dilution‐time profile of albumin was used to determine the volume kinetics of LRS infusion in conscious, healthy, euvolemic, adult horses. The data suggest rapid loss of the infused fluid from *V*_*c*_ (large *k*_12_), slow removal (low *k*_21_) from *V*_*p*_ and from the body (low *k*_10_) suggesting that a large proportion of rapidly administered IV fluid is retained in a peripheral compartment. Covariate analysis identified a negative influence of SAP on *k*_10_ and a positive influence of haemoglobin recruitment on *k*_12_. Fluid infusion led to increased heart rate and arterial blood pressure.

Teleologically, the slow turnover of fluid, which is slower when the arterial pressure is increased, is likely to increase the ability of the horse to endure periods of strenuous exercise without frequent ingestion of water. Slow fluid elimination in the clinical setting, however, offers no benefit but rather suggests that rapid or large volumes of fluid administration predispose horses to interstitial fluid accumulation and the potential for developing fluid overload. This emphasises the importance of correct dosing protocols and accurate measures of fluid responsiveness. Simulations based on VK parameters derived during various clinical conditions could provide fluid infusion protocols that optimise expansion of the central volume.

## FUNDING INFORMATION

This study was supported by the Fédération Equestre Internationale (FEI), HM King Hussein I Building, Chemin de la Joliette 8, 1006 Lausanne, Switzerland.

## CONFLICT OF INTEREST STATEMENT

The authors declare no conflicts of interest.

## AUTHOR CONTRIBUTIONS

**William W. Muir:** Conceptualization; data curation; formal analysis; writing – review and editing; funding acquisition; investigation. **Xiu Ting Yiew:** Writing – review and editing; software. **Shane W. Bateman:** Writing – review and editing; software. **Robert G. Hahn:** Software; methodology; writing – review and editing.

## DATA INTEGRITY STATEMENT

William W. Muir had full access to all the data in the study and takes responsibility for the integrity of the data and the accuracy of data analysis.

## ETHICAL ANIMAL RESEARCH

This study was approved by the Animal Care and Use Committee of Lincoln Memorial University and East Tennessee Clinical Research (ETCR) (protocol ID No. 23‐0314).

## INFORMED CONSENT

Not applicable.

## Supporting information


**Code S1.** Phoenix coding.

**Figure S1.** Biovenic Inc. Equine specific copeptin, ANP, aldosterone ELISA kits.

**Figure S2.** Urine output analysis.

**Table S1.** Akaike Information Criterion results for all models.

## Data Availability

The data that support the findings of this study are available upon reasonable request from the corresponding author. Open data sharing exemption granted by the editor.
