# Supplementary material for: Volume kinetics of lactated Ringer's solution in adult horses
Source: Equine Vet J. 2025 May 13;58(1):220–9. doi: 10.1111/evj.14534 (PMC12699107; doi:10.1111/evj.14534)
Supplement: Supplementary file 1 — Code S1. Phoenix coding. [file EVJ-58-220-s003.pdf]

Equine Veterinary Journal BEVA

Code S1:

Phoenix coding:

```
“Phoenix Build 8.3.5.340
Maximum Likelihood Models,  Version=8.3.5.340
------------------------------------
test(){
deriv(A1 = - Ke * A1 - K12 * A1 + K21 * A2)
deriv(A2 = K12 * A1 - K21 * A2)
urinecpt(A0 = Ke * A1)
dosepoint(A1)
C = A1 / V
error(CEps = 0.037263261745364)
observe(CObs = C + CEps)
error(A0Eps = 184.888123037488)
observe(A0Obs = A0 + A0Eps)
stparm(V = tvV * exp(nV))
stparm(Ke = tvKe * (SAP/mean(SAP))^dKedSAP * exp(nKe))
stparm(K12 = tvK12 * (1+(VcDerivSplenContr-median(VcDerivSplenContr))*dK12dVcDerivSplenContr) *
exp(nK12))
stparm(K21 = tvK21 * exp(nK21))
fcovariate(Bodyweight)
fcovariate(SAP)
fcovariate(DAP)
fcovariate(HR)
fcovariate(Temp)
fcovariate(RR)
fcovariate(FormDerivSplenContr)
fcovariate(VcDerivSplenContr)
fixef(tvV = c(, 24119.3219405964, ))
fixef(tvKe = c(, 0.00261231954916529, ))
fixef(tvK12 = c(, 0.0654321731598753, ))
fixef(tvK21 = c(, 0.00724312829787447, ))
fixef(dKedSAP(enable=c(0)) = c(, 0, ))
fixef(dK12dVcDerivSplenContr(enable=c(1)) = c(, 0.001, ))
ranef(diag(nV, nKe, nK12, nK21) = c(0.02389072, 0.82471347, 0.13558084, 0.59101371))
}
------------------------------------
id("ID")
time("Time")
dose(A1<-"Total_Volume", "Infusion_Rate")
covr(Bodyweight<-"Body_weight")
covr(SAP<-"SAP")
covr(DAP<-"DAP")
covr(HR<-"HR")
covr(Temp<-"Temp")
covr(RR<-"RR")
covr(FormDerivSplenContr<-"FormDeriv_Splen_Contr")
covr(VcDerivSplenContr<-"VcDeriv_Splen_Contr")
obs(CObs<-"Uncorrected_Alb_Dilution")
obs(A0Obs<-"Cumulative_Urine")
table(file="posthoc.csv", time(0), V,Ke,K12,K21)

------------------------------------
```

Run Options
Algorithm: FOCE ELS
N Iter:1000
Input sorted by subject+time
Enabling automatic log transform (if applicable)
ODE solver method: matrix exponent
Method of computing standard errors: Central Diff
Sandwich standard errors
Confidence Level %95
Maximum number of adaptive gaussian quadrature steps: 1
Simple run was performed"
