# Supplementary material for: Volume kinetics of lactated Ringer's solution in adult horses
Source: Equine Vet J. 2025 May 13;58(1):220–9. doi: 10.1111/evj.14534 (PMC12699107; doi:10.1111/evj.14534)
Supplement: Supplementary file 2 — Figure S1. Biovenic Inc. Equine specific copeptin, ANP, aldosterone ELISA kits. [file EVJ-58-220-s001.pdf]

Equine Veterinary Journal BEVA

Figure S1:
Biovenic Inc. Equine specific copeptin, ANP, Aldosterone ELISA kits:

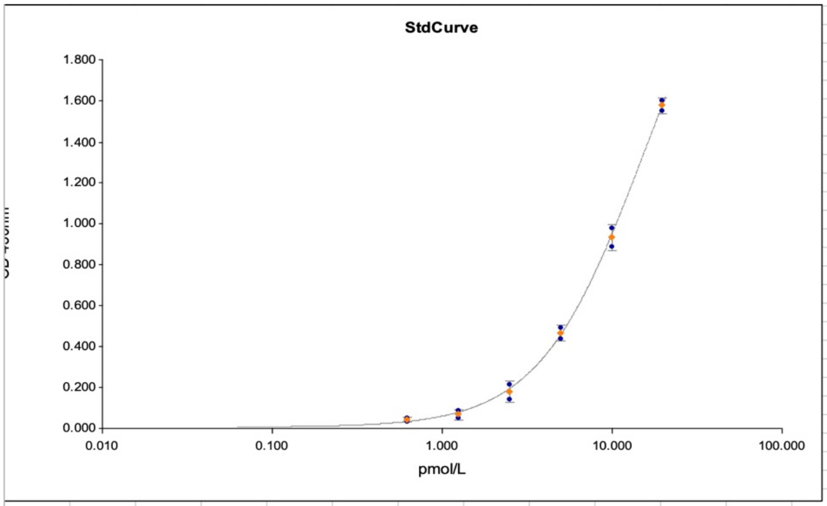

Curve Name	Curve Formula	Parameter	Value	Std. Error	95% CI min	95% CI max	Fit F Prob
StdCurve	Y = (A-D)/(1+(X/C)^B) + D	A	0.00609	0.0208	-0.0833	0.0955	?????
		B	1.43	0.135	0.846	2.01	
		C	15.2	2.61	4.01	26.5	
		D	2.64	0.316	1.28	4	

Curve Name	Curve Formula	A	B	C	D	R2	Fit F Prob
StdCurve	Y = (A-D)/(1+(X/C)^B) + D	0.00609	1.43	15.2	2.64	1	?????

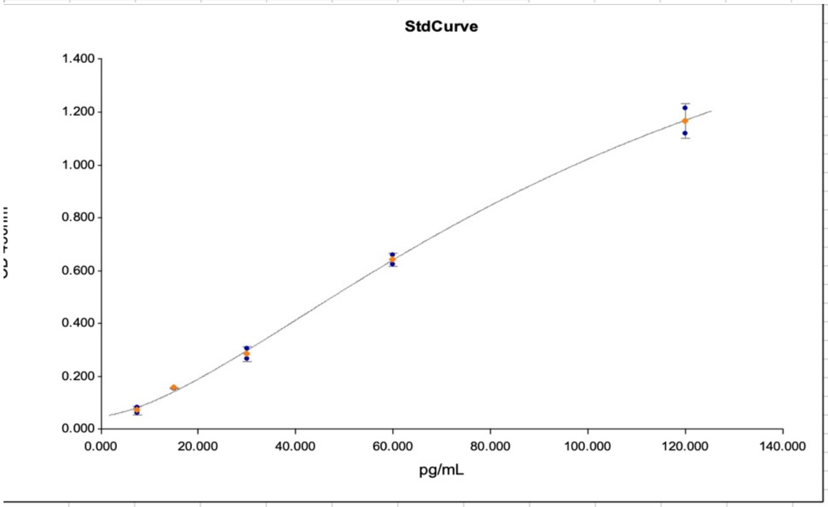

Curve Name	Curve Formula	Parameter	Value	Std. Error	95% CI min	95% CI max	Fit F Prob
StdCurve	Y = (A-D)/(1+(X/C)^B) + D	A	0.0488	0.0398	-0.456	0.554	?????
		B	1.56	0.389	-3.38	6.5	
		C	109	49.5	-520	738	
		D	2.13	0.751	-7.41	11.7	

Curve Name	Curve Formula	A	B	C	D	R2	Fit F Prob
StdCurve	Y = (A-D)/(1+(X/C)^B) + D	0.0488	1.56	109	2.13	0.999	?????

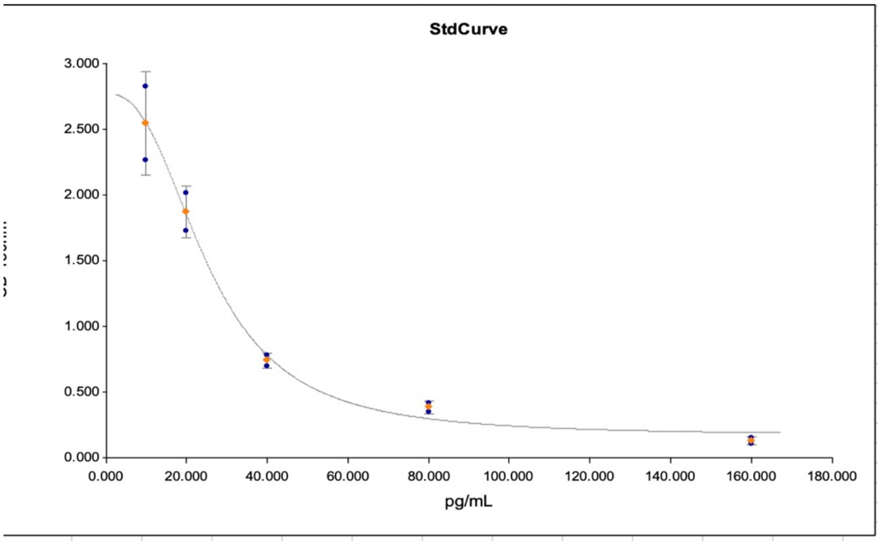

Curve Name	Curve Formula	Parameter	Value	Std. Error	95% CI min	95% CI max	Fit F Prob
StdCurve	Y = (A-D)/(1+(X/C)^B) + D	A	2.77	0.271	-0.671	6.21	?????
		B	2.58	0.757	-7.04	12.2	
		C	25.4	2.9	-11.5	62.2	
		D	0.17	0.126	-1.44	1.78	

Curve Name	Curve Formula	A	B	C	D	R2	Fit F Prob
StdCurve	Y = (A-D)/(1+(X/C)^B) + D	2.77	2.58	25.4	0.17	0.997	?????
