# Supplementary material for: Volume kinetics of lactated Ringer's solution in adult horses
Source: Equine Vet J. 2025 May 13;58(1):220–9. doi: 10.1111/evj.14534 (PMC12699107; doi:10.1111/evj.14534)
Supplement: Supplementary file 3 — Figure S2. Urine output analysis. [file EVJ-58-220-s004.pdf]

Equine Veterinary Journal BEVA

Figure S2: Urine output analysis.

Pop CWRES vs IVAR Final model

Panel A

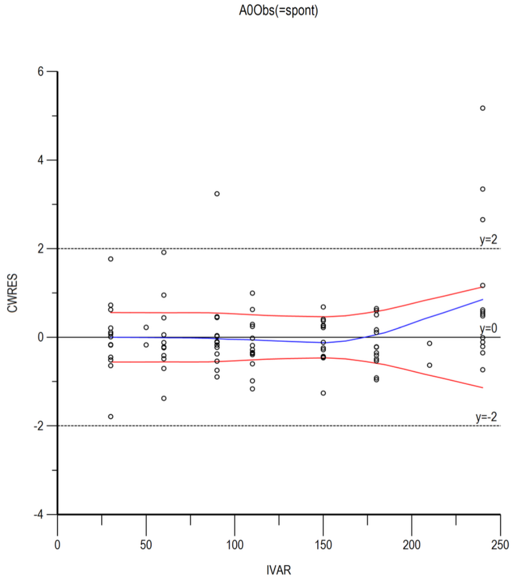

Panel B

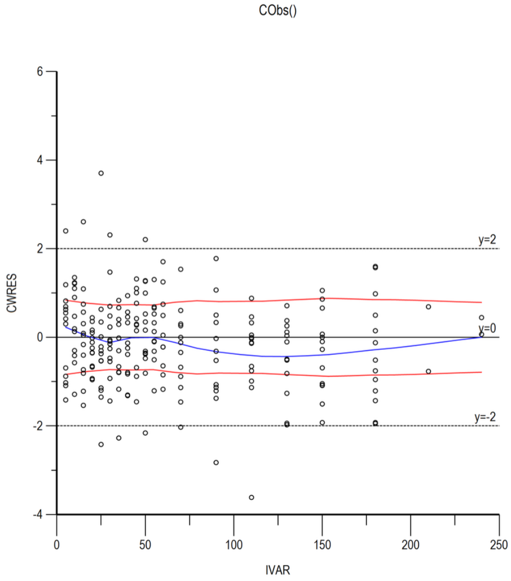

Panel A shows the conditional weighted residual values of the horse's urine output plotted against time. The parallel red lines that are equally distributed around midline and the blue line roughly paralleling zero line suggest that apart from the values from time point 240, that the model fit is very strong. There are 3 values outside of ± 3 that also indicate strong fit with the vast majority between ± 2. Panel B shows the conditional weighted residual values of the horse's plasma dilution plotted against time. The parallel red lines that are equally distributed around midline and the blue line roughly paralleling zero line suggest that the model fit is very strong. There are a handful of values from the first hour of the experiment that appear to have some minor influence on model fit. There are 2 values outside of ± 3 that also indicate strong fit with the vast majority between ± 2.

Pop CWRES vs PRED Final model

Panel A

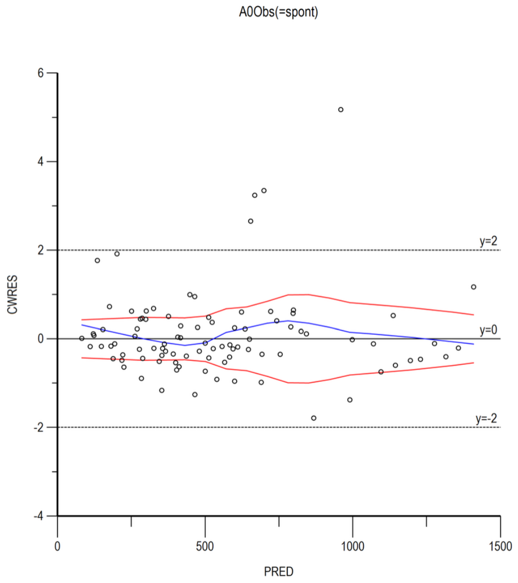

Panel B

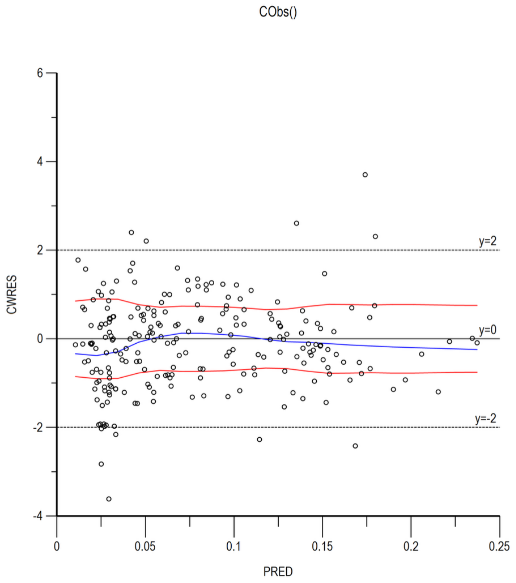

Panel A shows the conditional weighted residual values of the horse's urine output plotted against the population model-predicted urine output (random effects set to zero). The parallel red lines that are equally distributed around midline and the blue line roughly paralleling zero line suggest that the model fit is very strong. There are 3 values outside of ± 3 that also indicate strong fit with the vast majority between ± 2. Panel B shows the conditional weighted residual values of the horse's plasma dilution plotted against the population model-predicted plasma dilution (random effects set to zero). The parallel red lines that are equally distributed around midline and the blue line roughly paralleling zero line suggest that the model fit is very strong. There are 2 values outside of ± 3 that also indicate strong fit with the vast majority between ± 2.

Pop DV vs PRED Final Model

Panel A

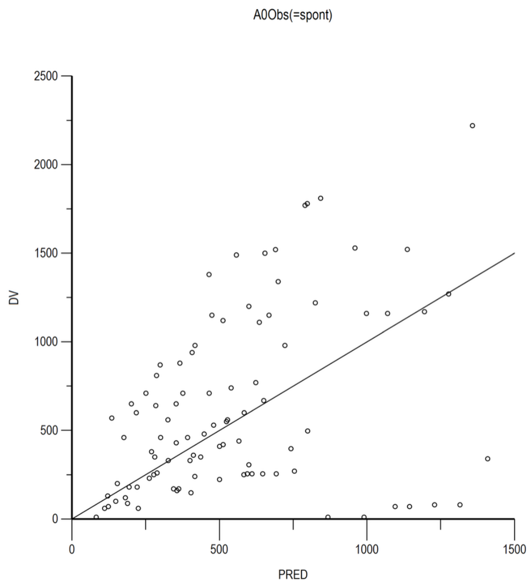

Panel B

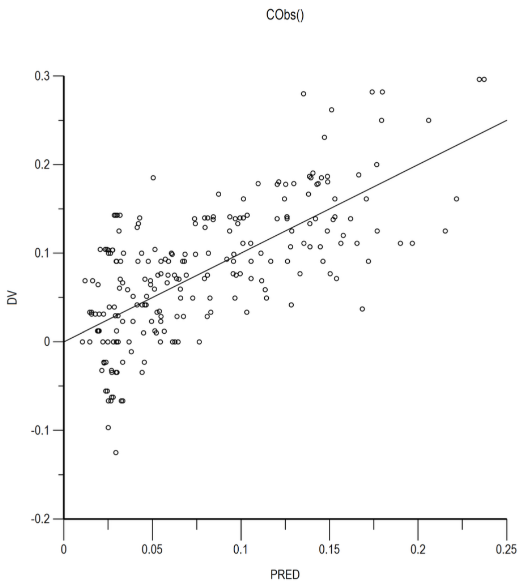

Panel A shows the horse's urine output plotted against the population model-predicted urine output (random effects set to zero). The data points should follow the line of unity but do not cluster around the line of unity as tightly when compared to individual model predicted values (where random effects are set to the empirical Bayesian estimate). Panel B shows the horse's plasma dilution plotted against the population model-predicted plasma dilution (random effects set to zero). The data points should follow the line of unity but do not cluster around the line of unity as tightly when compared to individual model predicted values (where random effects are set to the empirical Bayesian estimate).

Pop Visual Predictive Checks of Final Model1 (Panel A) and Pop Visual Predictive Checks of Final Model2 (Panel B)

Panel A

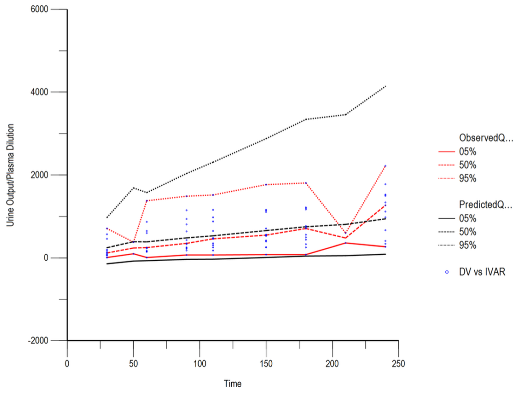

Panel B

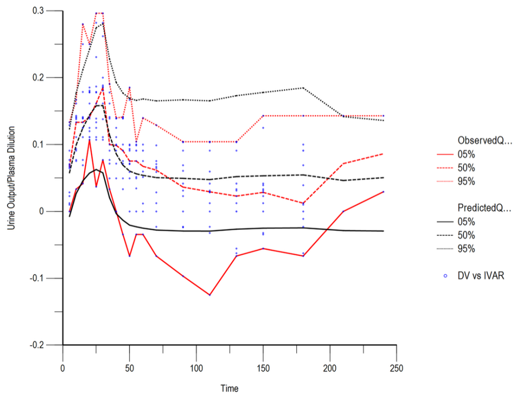

Panel A shows the Visual Predictive Checks of the observed urine output plotted against the model predicted values over time. The black lines represent the various plotted quartiles (5%, 50%, and 95%) for the model predicted values over time, and the red lines represent the various plotted quartiles (5%, 50%, and 95%) the actual patient values measured over time. For ideal model fit, the predicted quartile lines should tightly adhere to the actual values. In this case the 5% and 50% quartile plots are ideal, but the 95% quartile plot shows some deviation around the 45 and 210 minute time points. The plot indicates that the model is less accurate at predicting urine output in horses in the 95% quartile (high volume) of urine output. Panel B shows the Visual Predictive Checks plot of the observed plasma dilution plotted against the model predicted values over time. The black lines represent the various plotted quartiles (5%, 50%, and 95%) for the model predicted values over time, and the red lines represent the various plotted quartiles (5%, 50%, and 95%) the actual patient values measured over time. For ideal model fit, the predicted quartile lines should tightly adhere to the actual values. In this case the 50% quartile plots show strong model fit, but the 5% and 95% quartile plots indicate less effective model fit. The plots show a modest model fit, with more variation in model fit at the more extreme values in the 5% and 95% quartiles.
